# Cathepsin b: a potential prognostic marker for inflammatory breast cancer

**DOI:** 10.1186/1479-5876-9-1

**Published:** 2011-01-03

**Authors:** Mohamed A Nouh, Mona M Mohamed, Mohamed El-Shinawi, Mohamed A Shaalan, Dora Cavallo-Medved, Hussein M Khaled, Bonnie F Sloane

**Affiliations:** 1 Department of Pathology, National Cancer Institute, Cairo University, Giza 12613 Egypt; 2 Department of Zoology, Faculty of Science, Cairo University, Giza 12613 Egypt; 3 Department of General Surgery, Faculty of Medicine, Ain Shams University, Cairo 11566, Egypt; 4 Department of Surgery, National Cancer institute, Cairo University, Giza 12613 Egypt; 5 Department of Pharmacology, Wayne State University, Detroit, MI 48201, USA; 6 Department of Biological Sciences, University of Windsor, Windsor, ON, N9B 3P4 Canada; 7 Department of Medical Oncology, National Cancer Institute, Cairo University, Giza 12613 Egypt; 8 Barbara Ann Karmanos Cancer Institute, Wayne State University, Detroit, MI 48201, USA

## Abstract

**Background:**

Inflammatory breast cancer (IBC) is the most aggressive form of breast cancer. In non-IBC, the cysteine protease cathepsin B (CTSB) is known to be involved in cancer progression and invasion; however, very little is known about its role in IBC.

**Methods:**

In this study, we enrolled 23 IBC and 27 non-IBC patients. All patient tissues used for analysis were from untreated patients. Using immunohistochemistry and immunoblotting, we assessed the levels of expression of CTSB in IBC versus non-IBC patient tissues. Previously, we found that CTSB is localized to caveolar membrane microdomains in cancer cell lines including IBC, and therefore, we also examined the expression of caveolin-1 (cav-1), a structural protein of caveolae in IBC versus non-IBC tissues. In addition, we tested the correlation between the expression of CTSB and cav-1 and the number of positive metastatic lymph nodes in both patient groups.

**Results:**

Our results revealed that CTSB and cav-1 were overexpressed in IBC as compared to non-IBC tissues. Moreover, there was a significant positive correlation between the expression of CTSB and the number of positive metastatic lymph nodes in IBC.

**Conclusions:**

CTSB may initiate proteolytic pathways crucial for IBC invasion. Thus, our data demonstrate that CTSB may be a potential prognostic marker for lymph node metastasis in IBC.

## Background

Inflammatory breast cancer (IBC) is the most lethal form of primary breast cancer, with a 3-year survival rate of 40% as compared to 85% for non-IBC [1]. IBC is defined by distinct clinical features including a rapid onset, erythema, edema of the breast and a "peau d'orange" appearance of the skin. High metastatic behavior (for review see [2]), rapid invasion into blood and lymphatic vessels and formation of tumor emboli within these vessels [3] are also major characteristics of IBC. Obstruction of lymphatic flow by tumor emboli within the dermal lymphatics causes swelling of the breast tissue and underlies the inflammatory nature of the disease[3].

Positive axillary lymph node metastasis is a characteristic of IBC at the time of diagnosis and most IBC patients present with extensive lymph node metastasis [3,4]. Indeed, the number of positive metastatic lymph nodes contributes to poor survival outcome with each positive lymph node increasing risk of breast cancer mortality by approximately 6% [5]. Although IBC is characterized by the extensive presentation of metastatic lymph nodes, the molecular pathways that direct IBC lymph node invasion are not well defined. Recent studies conducted by Ellsworth and colleagues, using laser capture microdissection and gene expression analysis of primary breast tumors and corresponding metastatic lymph nodes, indicate that overexpression of genes involved in degradation of the extracellular matrix (ECM) in primary breast cancer cells induces them to disseminate to nearby lymph nodes [6].

The invasive properties of IBC are consistent with a crucial role for proteolytic enzymes in the degradation of ECM, cell motility and metastasis [7]. Cathepsin B (CTSB), a lysosomal cysteine protease, has been shown to be a contributor to the progression and invasion of various types of cancer [8]. Specifically, CTSB is involved in proteolytic pathways that lead to the degradation of ECM proteins thereby promoting cancer cell motility and invasion [8,9]. In cancer cells, CTSB is shuttled to the plasma membrane where it can activate receptor-bound pro-urokinase-type plasminogen activator (pro-uPA). uPA activate plasminogen a serine protease that can digest ECM proteins and activate MMPs, a family of proteolytic enzymes that are also major participants in ECM degradation and cancer cell motility and invasion [10]. CTSB is associated with cell surface caveolae, specialized membrane microdomains that are involved in signaling pathways, endocytosis and proteolysis (for review see [11,12]). The role of caveolin-1 (cav-1), the main structural protein of caveolae, in cancer progression and invasion is contradictory and appears to depend upon the cancer type and stage of progression. In IBC patient tissues and cell lines, cav-1 is overexpressed [7], a phenotype observed in other aggressive breast carcinomas that show high metaplastic properties [13]. Overexpression of cav-1 has been shown to be associated with ECM degradation and formation of invadopodia, which contain membrane-type-1-MMP (MT1-MMP) and mediate breast cancer cell motility and invasion [14]. In previous *in vitro *studies, we have shown that interaction of IBC cells with human monocytes augments invasion of IBC cells through increased ECM degradation, events correlated with an increase in CTSB expression, secretion and activity and an increase in cav-1 expression in the IBC cells [15]. More recently, we have co-localized active CTSB and uPA with cav-1 in caveolar fractions of SUM149 IBC cells (unpublished data).

In the present study, we assessed the expression levels of CTSB and cav-1 in IBC versus non-IBC patient breast tissues. Furthermore, we examined the correlation between these proteins and the number of metastatic lymph nodes in IBC versus non-IBC patient tissues. Our results revealed an overexpression of CTSB and cav-1 in IBC tissues and demonstrated a positive correlation between CTSB expression and the number of positive lymph node metastases. We speculate that CTSB expressed by tumor cells and localized in caveolae may promote IBC metastasis to lymph nodes by enhancing ECM degradation and tumor invasion.

## Methods

### Patients and Tissue Specimens

**Figure 1:**
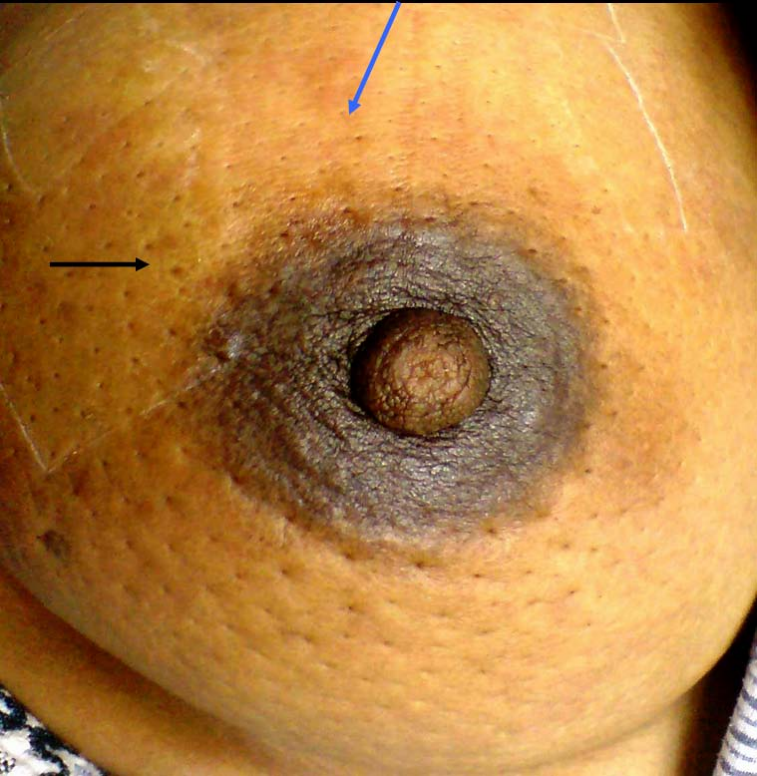
**Photograph of IBC patient showing clinical criteria for IBC diagnosis, i.e., edema, erythema (blue arrow) and peau d'orange (black arrow)**.

For the purpose of patient enrollment in this study, we obtained Institutional Review Board (IRB) approval from the ethics committee of Ain-Shams University and the National Cancer Institute (NCI), Cairo University. Patients were selected from those referred to outpatient breast clinics of Ain Shams University hospital and NCI Cairo University during the period of June 2008 to December 2009. Inclusion criteria of breast cancer patients were dependent upon a combination of clinical, mammographic, ultrasound, and pathological diagnoses. Clinical diagnosis of IBC is applied, according to the American Joint Committee on Cancer (AJCC) T4 d designation for IBC (for review see [16]), when a patient presented with a diffuse erythema, peau d'orange and edema of the breast (Figure 1). For IBC patients, pathological confirmation of the clinical diagnosis was dependent upon examination of both skin and core biopsies (M.A.N.). In the absence of breast masses, diagnosis was depended upon pathological examination of skin biopsies that showed permeation of dermal lymphatics by carcinoma cells and the presence of dermal tumor emboli (M.A.N.). Non-IBC patients of stage II-III were also included in our study as a comparison group. Patients subjected to neo-adjuvant chemotherapy or those with viral hepatitis or autoimmune disease were excluded from our study. Based on the criteria described here, we enrolled 23 IBC and 27 non-IBC patients in the present study.

Tissue samples were fixed in 10% neutral buffered formalin and processed into paraffin blocks for routine sectioning and immunohistochemistry (IHC). Pathological data regarding tumor size, tumor grade [17], and the presence of lymphovascular invasion, dermal tumor emboli and tumor parenchyma emboli [2,18] were assessed (M.A.N), reviewed (H.I.) and tabulated for statistical analysis. Additional sections were generated from the paraffin tissue blocks and immunostained for estrogen receptor (ER), progesterone receptor (PR) and HER2-neu expression status. IHC staining for CTSB, and cav-1 was performed as described below.

### Immunohistochemistry

Mouse anti-caveolin-1 was purchased from BD Biosciences (San Diego, CA, USA) and polyclonal rabbit anti-human CTSB antibody was previously prepared in house (B.F.S.) [19]. Antibody diluent with background reducing components and DakoCytomation EnVision+ Dual Link System-HRP (DAB+) kits were purchased from Dako (Carpinteria, CA, USA); and Permount^® ^was from Fisher Scientific (Pittsburgh, PA, USA).

Tissue sections were prepared from paraffin blocks and stained with hematoxylin and eosin to select tissue sections for immunostaining and scoring. IHC staining for each marker was performed in duplicate on 5 μm thick tissue sections. Tissue sections were first deparaffinized and rehydrated followed by antigen retrieval. Tissue sections were incubated for 1 hour at room temperature with the following primary antibodies prepared in Dako Antibody diluent with reduced background components: polyclonal CTSB antibody (1:500) and monoclonal anti-cav-1 (1:150). Detection was carried out by incubating tissue sections with 100 μl of horse radish peroxidase-labeled rabbit or mouse secondary antibody [EnVision+ Dual Link System-HRP (DAB+)] for 45 min. Staining was achieved by adding 100 μl of DAB+ diluted 1:50 in substrate buffer [EnVision+ Dual Link System-HRP (DAB+)] for 15 min. Nuclei were counterstained with hematoxylin and specimens were rinsed in PBS and mounted using Permount^® ^for microscopic examination. Negative control slides were run in parallel in which each primary antibody was replaced with PBS.

Two independent readers (M.A.N. and M.M.M.) assessed immunostaining of CTSB and cav-1 using light microscopy (Olympus, CX41, Japan). Discordant results were resolved by consultation with a third reader (H.I.). The expression of CTSB B and cav-1 was scored according to both the intensity of staining and the proportion of positive staining carcinoma cells within the entire slide: "0", no immunostaining was observed within carcinoma cells; "+", less than 10% of carcinoma cells showed cytoplasmic staining of moderate to marked intensity; "++", 10-50% of carcinoma cells showed cytoplasmic staining of moderate to marked intensity; and "+++", greater than 50% of carcinoma cells show cytoplasmic staining of moderate to marked intensity.

### SDS-Polyacrylamide Gel Electrophoresis (PAGE) and Immunoblotting

Peroxidase-labeled goat anti-rabbit secondary antibody and tetramethyl benzidine (TMB membrane peroxidase substrate were purchased from Kirkegaard and Perry Laboratories Inc (Gaithersburg, MD, USA).

Fresh breast tissue specimen obtained from core biopsy or during modified radical mastectomy were minced into small pieces on ice in RIPA buffer [25 mM Tris-HCl pH 7.6, 150 mM NaCl, 1% NP-40, 1% sodium deoxycholate, 0.1% SDS (Sigma-Aldrich, St. Louis, MO, USA)]. Protein concentrations of cell lysates were measured using Bradford reagent (Sigma-Aldrich, Germany). Samples were equally loaded (20 μg protein/well), separated by 12% SDS-PAGE under reducing conditions and transferred onto nitrocellulose membranes as previously described [20]. Immunoblotting analysis was performed using primary antibodies against CTSB (1:4000) and caveolin-1 (1:5000) and a secondary antibody conjugated with horseradish peroxidase (1:10,000) in Tris-buffered saline wash buffer (20 mM Tris, pH 7.5, 0.5 M NaCl) containing 0.5% Tween 20 and 5% (w/v) non-fat dry milk. After washing, bound antibodies were detected by adding a TMB chromagen/substrate solution. Once a signal was detected reactions were terminated by immersing membranes in water for 20-30 seconds.

### Statistical Analysis

The data were analyzed using SPSS software version 16.0. Differences were evaluated by Student's t-test and Fisher's exact test. Immunohistochemical scores of 0 and + were considered negative and scores of ++ and +++ were considered positive. Fisher exact test was performed to analyze differences in CTSB and cav-1 immunostaining (i.e., positive versus negative) between IBC and non-IBC groups. Correlations between categorical variables were assessed using Fisher's exact test as previously described [21].

## Results

### Clinical and pathological characterization of IBC versus non-IBC patients

Clinical and pathological characterization of the IBC (n = 23) and non-IBC patients (n = 27) used in this study is indicated in Table 1. Age of IBC patients ranged from 29-60 years (mean age of 40.9 ± 7.5), whereas the age of non-IBC patients ranged from 33-67 years (median age of 49.9 ± 9.1 Thus, IBC patients were significantly (*P *= 0.001) younger at the time of diagnosis as compared to non-IBC patients.

**Table 1:** Clinical and pathological characterization of IBC versus non-IBC patients

Clinical characteristic	IBC *n *= 23 (%)	Non-IBC *n *= 27 (%)	p-value
**Age**			
Range	29-60	33-67	0.001^a^*
Mean ± SD	40.9 ± 7.5	49.9 ± 9.1	t- test

**Tumor size‡**			
Mean ± SD	6.5 ± 3.3	4.31 ± 2.30	1.000^b^
< 2	1 (5.6%)	1 (3.7%)	
≥ 2	17 (94.4%)	26 (96.3%)	

**Tumor grade**			
I- II	15 (65%)	21 (77.8%)	0.511^b^
III	8(35%)	6 (22.2%)	

**Axillary Lymph Node Status†**			
Negative	0(0%)	7 (25.9%)	0.037^b^*
< 4	3 (15%)	9 (33.4%)	
4-7	6 (30%)	6 (22.2%)	
≥ 8	11(55%)	5 (18.5%)	

**ER**			
Positive	6 (27.3%)	6 (22.2%)	
Negative	17 (72.7%)	21 (77.8%)	0.747^b^

**PR**			
Positive	7 (31.8%)	8 (29.6%)	1.000^b^
Negative	16 (68.2%)	19 (70.4%)	

**HER-2**			
Positive	4 (18.2%)	4 (14.8%)	1.000^b^
Negative	19 (81.8%)	23 (85.2%)	

**Lymphovascular invasion**			
Positive	17 (73.9%)	3 (11.1%)	0.000^b^*
Negative	6 (26.1%)	24 (88.9%)	

**Tumor emboli**			
Positive	23 (100%)	3 (11.1%)	0.000^b^*
Negative	0	24 (88.9%)	

* Significant p value calculated by ^a^Student- T test or ^b^Fisher's exactTest

**‡ ***n *= 18 (five IBC patients did not have a tumor mass)

**† ***n *= 20 (three patients were not evaluated because they died before surgery)

Tumor size measurements revealed that 5 IBC patients (21.7%) presented with no tumor mass that could be detected clinically, mammographically or upon examination of the mastectomy specimen; however, tumor emboli were present in skin and core biopsies. For IBC patients with detectable masses, 5.6% of them exhibited tumor masses less than 2 cm and 94.4% had a tumor mass more than 2 cm with tumor sizes ranging from 4-10 cm (mean size of 6.5 ± 3.3 cm). Non-IBC patients had tumor sizes ranging from 1.8-12 cm (mean size of 4.3 ± 2.3 cm) with 3.7% having tumor sizes less than 2 cm and 96.3% having tumor sizes greater than or equal to 2 cm.

Tumor grading revealed that 65% of IBC patients were tumor grade I or II and 35% were tumor grade III. In non-IBC patients 77.8% were diagnosed as tumor grade I or II, and 22.2% were diagnosed as tumor grade III.

We assessed the number of axillary lymph nodes that were positive for metastases in IBC versus non-IBC patients. All IBC patients who underwent surgery had positive metastatic lymph nodes: 15% had 1-3 positive metastatic lymph nodes, 30% had 4-7 positive metastatic lymph nodes and 55% had greater than or equal to 8 positive metastatic lymph nodes. Among non-IBC patients, 25.9% were node negative, 33.4% had 1-3 metastatic lymph nodes, 22.2% had 4-7 metastatic lymph nodes and 18.5% had greater than or equal to 8 positive metastatic lymph nodes. In addition, the difference between the number of positive metastatic lymph nodes in IBC versus non-IBC patients was determined to be statistically significant (*P *= 0.037).

Lymphovascular invasion was significantly greater (*P *= 0.000) in IBC (73.9%) versus non-IBC (11.1%) patients. Tumor emboli, a phenotypic hallmark of IBC and defined as tight tumor cell clusters retracted away from the surrounding endothelial lining [2,18], were detected in 100% of IBC tissue sections as compared to only 11.1% of non-IBC tissue sections (*P *= 0.000). Positive staining for ER, PR and HER-2 was detected in 27.3%, 31.8% and 18.2% of the IBC patients, respectively. In non-IBC patients, positive staining for ER, PR and HER-2 was 22.2%, 29.6% and 14.8%, respectively.

### Overexpression of CTSB in IBC versus non-IBC tissues

**Figure 2:**
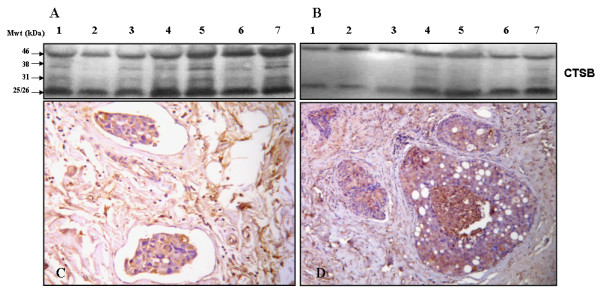
**CTSB expression in IBC versus non-IBC tissues**. [A] Expression of CTSB in IBC tissue homogenates from 7 different patients (lanes 1-7) was determined by immunoblotting. The forms of CTSB detected were the proenzyme (46 kDa), an intermediate form (38 kDa), single chain mature enzyme (31 kDa) and the heavy chain of double chain mature enzyme (25/26 kDa). β-actin was used as a loading control. [B] Tumor lymphatic emboli in IBC tissue sections, showing CTSB immunostaining (magnification X400). [C] Expression of CTSB in non-IBC tissue homogenates from 7 different patients (lanes 1-7) by immunoblotting analysis. [D] Immunostaining for CTSB in non-IBC tissue (magnification X400).

To assess the level of expression of CTSB in tissue homogenates of IBC versus non-IBC patients, we used immunoblotting analysis. Results showed that different forms of CTSB comprising pro-CTSB (46-kDa); intermediate-CTSB (38 kDa); and mature-CTSB forms (31 kDa single chain and 25/26 kDa double chain) were highly expressed in IBC tissues (Figure 2A) as compared to non-IBC tissues (Figure 2B).

To further localize cellular expression of CTSB in IBC versus non-IBC carcinoma cells, we used IHC to stain CTSB in paraffin embedded tissue sections. Results of IHC staining were scored for the intensity of CTSB staining (Table 2). CTSB was localized in the cytoplasm and cell membrane of IBC tumor emboli (Figure 2C) and non-IBC carcinoma cells (Figure 2D).

**Table 2:** Scoring of CTSB and cav-1 expression in breast carcinoma cells in IBC versus non-IBC tissues

	CTSB		Cav-1	
	
	IBC	Non-IBC	IBC	Non-IBC
	
	*n *(%)	*n *(%)	*n *(%)	*n *(%)
negative	0 (0%)	1 (3.7%)	0 (0%)	13 (48.2%)

**+**	0(0%)	5 (18.5%)	7 (30.4%)	8 (29.6%)

**++**	8 (34.8%)	7 (25.9%)	9 (39.2%)	2 (7.4%)

**+++**	15 (65.2%)	14 (51.9%)	7 (30.4%)	4 (14.8%)

Fisher's exact test	*P *= 0.025*		*P *= 0.001*	

n: number of patients

* Significant *P *value

IHC scoring results revealed a statistical significance (*P *= 0.025) in the level of expression of CTSB in IBC versus non-IBC carcinoma cells. In IBC, 34.8% showed CTSB staining score of ++ and 65.2% showed staining score of +++. In non-IBC, CTSB staining was variable with 3.7% scoring 0, 18.5% scoring +, 25.9% scoring ++ and 51.9% scoring +++ (Table 2).

### Overexpression of cav-1 in IBC versus non-IBC tissues

**Figure 3:**
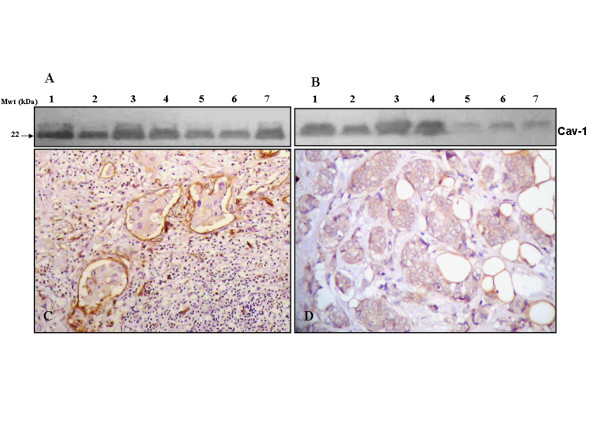
**Cav-1 expression in IBC versus non-IBC tissues**. [A] Immunoblot analysis showing expression of cav-1 (22 kDa) in IBC tissue homogenates from 7 different patients (lanes 1-7). [B] Tumor lymphatic emboli in IBC tissue sections showing expression of cav-1 (magnification X400) [C] Cav-1 level of expression in non-IBC tissue homogenates from 7 different patients (lanes 1-7). [D] Non-IBC invasive ductal carcinoma showing expression of cav-1 in breast carcinoma cells (magnification X200).

Immunoblot analysis revealed an overexpression of cav-1 (22 kDa) in IBC tissues as compared to non-IBC tissues (Figure 3A and 3B). Using IHC staining, we showed that 100% of IBC tissues express cav-1 (Figure 3C) whereas only 51.8% of non-IBC samples expressed cav-1 (Figure 3D). Scoring for cav-1 expression in IBC (Figure 3C) cells was as follows: 30.4% scored +, 39.2% scored ++ and 30.4% scored +++ (Table 2). In the non-IBC tissues (Figure 3D), 48.2% of patient tissue samples revealed negative staining for cav-1 in carcinoma cells, whereas 29.6% scored +, 7.4% scored ++ and 14.8% scored +++ (Table 2). Our results revealed a statistically significant overexpression of cav-1 (*P *= 0.001) in IBC versus non-IBC patients. The present results agree with those of Van den Eynden et al. [7] in demonstrating an overexpression of cav-1 in IBC patient tissues.

### Expression of CTSB correlates with positive metastatic lymph nodes in IBC

We tested whether the number of positive metastatic lymph nodes correlates with the expression levels of each of CTSB and cav-1 in IBC versus non-IBC patient tissues. In the IBC patient group, CTSB showed a statistically significant correlation (*P *= 0.0478) with the presence of positive metastatic lymph nodes as compared to the non-IBC group (Table 3). Cav-1 expression showed statistically non-significant correlation (*P *= 0.0717-this number does not match table 3) with the number of positive lymph node metastasis (Table 3).

**Table 3:** Correlation between lymph node metastasis and expression of CTSB and cav-1 in IBC versus non-IBC patients.

Variable	CTSB Expression		Cav-1 Expression	
	IBC (%)	Non-IBC (%)	IBC (%)	Non-IBC (%)
Lymph node metastasis				
Negative	0 (0%)	5 (23.8%)	0 (0%)	3 (27.2%)
Positive	20 (100%)	16 (76.25)	14 (100%)	8 (72.7%)
Fisher's exact test	*P *= 0.0478*		*P *= 0.0717	

*Significant p value calculated by Fisher's exact test

Thus, our data reveal that the overexpression of CTSB in IBC versus non-IBC is significantly correlated with the increase in number of positive metastatic lymph nodes, suggesting a potential role for this proteolytic enzyme in promoting the invasion of IBC cells into lymphatic vessels.

## Discussion

Criteria for the TNM staging system for breast cancer indicate that the number of positive metastatic axillary lymph nodes is one of the most important prognostic factors for predicting a low survival rate of breast cancer patients [22]. Despite therapeutic regimes, patients with 10 or more positive lymph nodes have a 70% chance of disease recurrence [23,24]. Indeed, dissemination of IBC cells to lymph nodes is consistent with the aggressive phenotype of IBC although the molecular and cellular pathways underlining this process are poorly understood. In the present study, we show a significant positive correlation between expression of the cysteine protease CSTB and the number of metastatic lymph nodes in IBC patients. In addition, cav-1 was also shown to be overexpressed in IBC tissue as compared to non-IBC tissue.

Our previous *in vitro *studies showed that increased ECM degradation and invasion of the SUM149 IBC cell line are associated with an overexpression of CTSB and cav-1 [15]. Cav-1 is the main structural protein of lipid raft caveolae, a site that has been hypothesized to localize cell surface proteases involved in pericellular proteolytic events [12]. Indeed, downregulation of cav-1 in colorectal carcinoma cells decreased trafficking of CTSB to caveolae on the surface of these cells and decreased degradation of ECM proteins and cellular invasion [25]. Although the role of cav-1 in breast cancer is contradictory, overexpression of cav-1 is present in aggressive types of breast cancer such as metaplastic carcinoma [13] and IBC [7]. Moreover, in IBC cell lines and tissues, overexpression of cav-1 is correlated with increased RhoC expression, a GTPase involved in cell motility and invasion [7]. In the present study, overexpression of cav-1 did not significantly correlate with an increase in expression of CSTB; however, current studies in our laboratory have localized CTSB to caveolae of SUM149 IBC cells (unpublished data). Moreover these cells exhibit extracellular degradation of ECM proteins that was partially blocked by cysteine and serine protease inhibitors (unpublished data). Thus, our data suggest that overexpression of cav-1 in IBC cells contributes to proteolytic events involving CTSB that lead to ECM degradation, tumor invasion and metastasis.

IBC is characterized by extensive involvement of positive metastatic lymph nodes, which are associated with the aggressive phenotype of the disease [26] and are a determining factor in therapeutic decisions [27-29]. As such, we determined whether there were correlations between CTSB and cav-1 and the number of positive metastatic lymph nodes in IBC versus non-IBC patients. Our results revealed a statistically significant positive correlation only between the level of CTSB expression in IBC carcinoma cells and the number of positive metastatic lymph nodes (*P *= 0.0478). Such a correlation was not detected in non-IBC patients. A positive correlation between CTSB expression and the metastasis of carcinoma cells to lymph nodes has previously been reported in breast [30], prostate [31] and gastric [32] cancers. Overexpression of CTSB in breast cancer has been shown to enhance tumor growth and invasion [33]. This parallels increased recurrence and shortened disease-free survival [30]. Moreover in an animal mammary cancer model, the number of positive metastatic lymph nodes has also been found to be associated with expression of CTSB [34]. Thus, our data are consistent with a crucial role for CTSB in promoting the highly metastatic behaviour of IBC.

## Conclusions

The positive correlation between CTSB and nodal metastatic burden in IBC patients suggests that this proteolytic enzyme may promote nodal metastasis in IBC patients. We hypothesize that the overexpression of cav-1 in IBC increases trafficking of CTSB to the cell surface where it promotes IBC invasion into lymphatic vessels and metastasis to lymph nodes. Further studies to validate CTSB as a prognostic marker in IBC and delineate the mechanisms by which the association of CTSB with cav-1 is involved in lymph node metastasis in IBC patients are in progress.

## Competing interests

The authors declare that they have no competing interests.

## Authors' contributions

All authors read and approved the final manuscript

B.F.S., M.M.M. and D.C.M. were responsible for the design of the study and critical revisions of the manuscript. M.A.N. was responsible for patients' pathological evaluation, performing IHC and scoring analysis. M.M.M. was responsible for conducting laboratory experimental procedures, their interpretation and manuscript preparation. M.E.S. was responsible for patients' recruitment, clinical diagnosis, patients' follow-up, providing patients' data and contributions to manuscript preparation. M.A.S. participated in patients' recruitment. H.M.K was responsible for patients' treatment decisions, participated in scientific discussions and revision of the manuscript.
